# P-754. Retrospective Study of Necrotizing Fasciitis: A Review of 99 Cases in a Tertiary Care Center in Eastern Thailand from 2022 to 2023

**DOI:** 10.1093/ofid/ofae631.950

**Published:** 2025-01-29

**Authors:** Tuntanut Lohawatcharagul, Supaschin Jamjittrong, Thanathip Suenghataiphorn, Arp-Arpa Kasemsantitham, Ponphisudti Tangsirisatian, Sarita Rojasavastera, Tinn Hongboontry, Tunyaporn Kamonvarapitak, Nuttarak Sasipong, Sichon Luerithiphong

**Affiliations:** Faculty of Medicine, Chulalongkorn University, Bangkok, Krung Thep, Thailand; Queen Savang Vadhana Memorial Hospital, Si racha, Chon Buri, Thailand; Griffin Hospital, Ansonia, Connecticut; Faculty of Medicine, Chulalongkorn University, Bangkok, Krung Thep, Thailand; Faculty of Medicine, Chulalongkorn Hospital, Bangkok, Thailand, Bangkok, Krung Thep, Thailand; Faculty of Medicine, Chulalongkorn University, Bangkok, Krung Thep, Thailand; Faculty of Medicine, Chulalongkorn University, Bangkok, Krung Thep, Thailand; Queen Savang Vadhana Memorial Hospital, Si racha, Chon Buri, Thailand; Faculty of Medicine, Chulalongkorn Hospital, Bangkok, Thailand, Bangkok, Krung Thep, Thailand; Faculty of Medicine, Siriraj Hospital, Bangkok, Krung Thep, Thailand

## Abstract

**Background:**

Necrotizing fasciitis (NF) is a rare and fatal soft tissue infection that requires immediate diagnosis and management. Data on microbiological aspects, clinical features, and outcomes of NF remain unexplored, particularly in certain global regions. With its high mortality rate, comprehensive understanding of NF is crucial.

**Figure 1.**
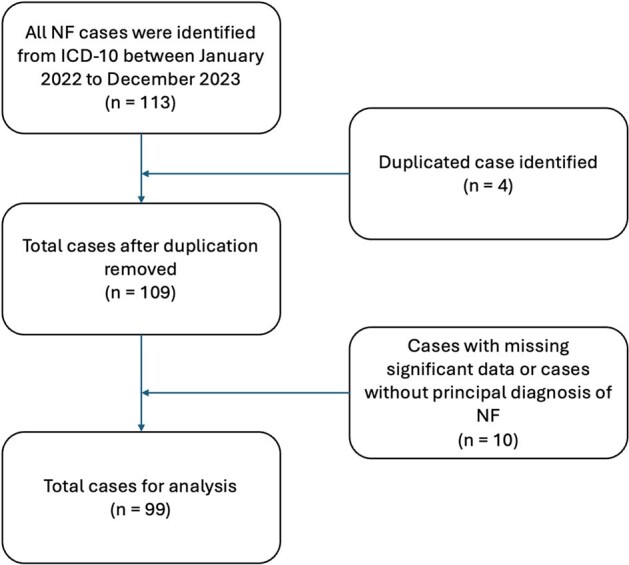
Inclusion Flow Diagram of Patients Diagnosed with Necrotizing Fasciitis between 2022-2023

**Methods:**

All NF cases diagnosed at a tertiary care center in Eastern Thailand between January 2022 - December 2023 were retrospectively reviewed. Patient’s demographic, comorbidities, clinical presentations, laboratory data, treatment modalities, and clinical outcomes were evaluated.

**Table 1.**
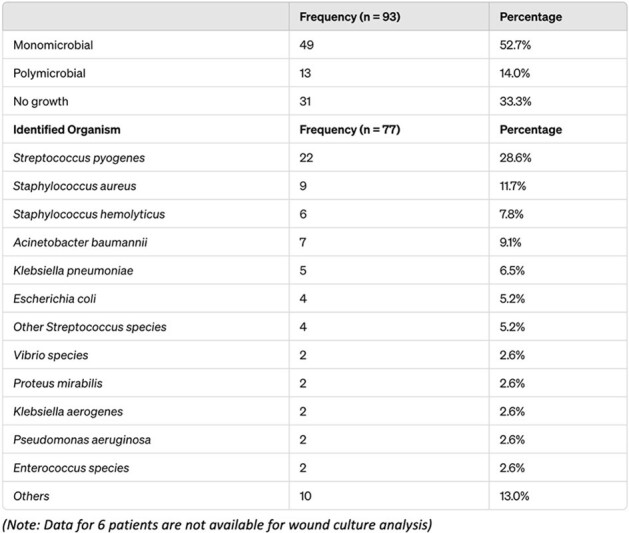
Microbial Profile and Organism Distribution in Necrotizing Fasciitis Cases

**Results:**

Among 99 NF cases identified, the mean age was 54.85 years (SD = 13.5), and 52.5% were males. Most common comorbidities were hypertension (48.5%), diabetes (44.4%), and dyslipidemia (38.4%). Wound cultures revealed 52.7% monomicrobial, 14% polymicrobial, and 33.3% no growth, with *Streptococcus pyogenes* being the most prevalent (28.6%). Average number of debridements per case was 3.06, and 53.5% of the patients underwent vacuum dressing with an average duration of 24.07 days (SD = 18.8). Significantly fewer patients underwent debridement within 24 hours if the initial diagnosis was cellulitis prior to being diagnosed with NF (21/43 (48%) versus 44/56 (78.5%), p = 0.002). The most common antibiotic regimen was ceftriaxone plus clindamycin (78.8%). Mortality occurred in 8 cases (8.08%), while 11 cases (11.1%) had amputations and 26 cases (26.3%) experienced severe sepsis.

**Table 2.**
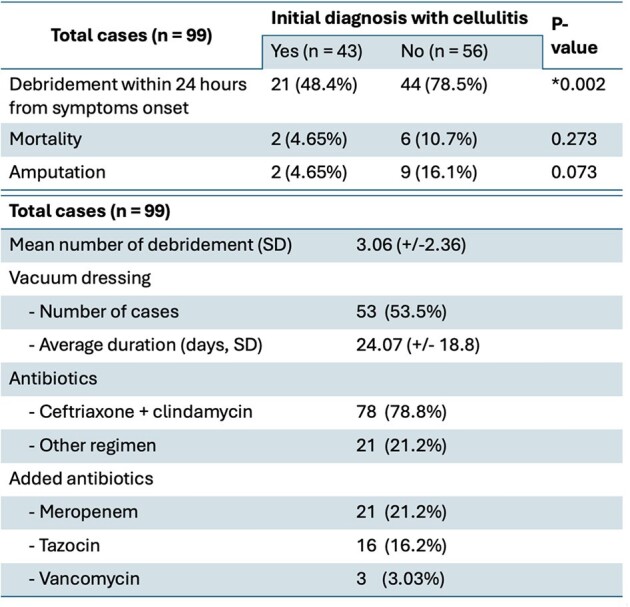
Treatment Modalities and Their Attributes in Patients Diagnosed with Necrotizing Fasciitis

**Conclusion:**

Our cohort exhibits a notably low mortality rate, compared to existing literature. Here, we present the first study exploring NF characteristics in Eastern Thailand. With findings indicating delayed debridement among patients initially diagnosed with cellulitis, this emphasizes the significance for prompt diagnosis of NF.

**Table 3.**
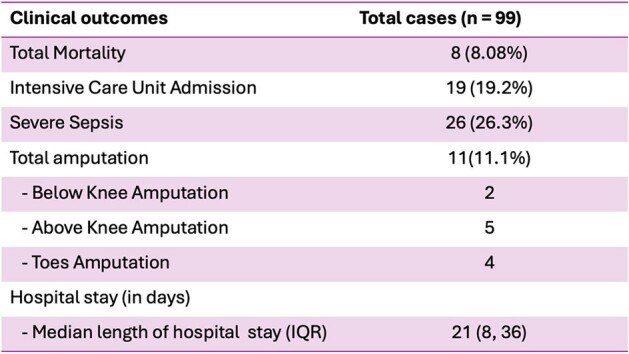
Clinical Outcomes in Study Cohort of 99 patients with Necrotizing Fasciitis

**Disclosures:**

**All Authors**: No reported disclosures

